# The effect of oral curcumin capsules on symptoms of premenstrual syndrome and dysmenorrhea: a randomized controlled trial

**DOI:** 10.1186/s40780-025-00470-1

**Published:** 2025-07-16

**Authors:** Fatemeh Shabani, Sepideh Mashayekh-Amiri, Fatemeh Teihoomaneshfard, Mahsa Hesami, Elnaz Shaseb, Mojgan Mirghafourvand

**Affiliations:** 1 Midwifery Department, Faculty of Nursing and Midwifery, Tabriz University of medical Sciences, Tabriz, Iran; 2 Counselling and Reproductive Health Research Centre, Department of Midwifery, Faculty of Nursing and Midwifery, Golestan University of Medical Sciences, Gorgan, Iran; 3 Department of Clinical Pharmacy, Faculty of Pharmacy, Tabriz University of Medical Sciences, Tabriz, Iran; 4 Social Determinants of Health Research Center, Faculty of Nursing and Midwifery, Tabriz University of Medical Sciences, Tabriz, Iran

**Keywords:** Curcumin, Premenstrual syndrome, Dysmenorrhea

## Abstract

**Background:**

Given the high prevalence of menstrual disorders such as premenstrual syndrome (PMS) and dysmenorrhea among students and the anti-inflammatory and antioxidant properties of curcumin, we decided to conduct a study to determine the effects of curcumin on PMS and dysmenorrhea among medical students in Tabriz-Iran.

**Methods:**

This double-blind randomized controlled trial was conducted on 62 students from Tabriz University of Medical Sciences in Iran during the 2023–2024 academic year. The participants were randomly assigned to the intervention (*n* = 31) and control (*n* = 31) groups. They were given curcumin and placebo capsules with the same dose of 500 mg once daily for 10 days during each of the two menstrual cycles (7 days before to 3 days after the estimated onset of menstruation). Data collection involved a sociodemographic characteristics questionnaire, the Premenstrual Symptoms Screening Tool (PSST), the Visual Analogue Scale (VAS), and a checklist to evaluate potential side effects. Independent t test and ANCOVA were employed to compare the results between the two groups.

**Results:**

There was no significant difference between the study groups regarding sociodemographic characteristics and baseline values before the intervention. Based on the ANCOVA test and by adjusting the baseline values, the curcumin group showed a significant reduction in average score of PSST in 2nd menstrual cycle (MD: -5.2; 95% CI: -9.6 to -0.9; *P* = 0.018) and average score of VAS in 2nd menstrual cycle (MD: -0.8; 95% CI: -1.4 to -0.1; *P* = 0.012) and 3rd menstrual cycle (MD: -0.8; 95% CI: -1.5 to -0.08; *P* = 0.029) compared to the placebo group. None of the study groups reported adverse effects. There were no dropouts and all participants completed the treatment period successfully.

**Conclusion:**

This study highlights the increasing evidence that curcumin is an effective natural treatment for PMS and dysmenorrhea. Further research into dose optimization, combination therapies, and long-term effects will strengthen its position in clinical practice.

**Trial registration:**

Iranian Registry of Clinical Trials (IRCT) IRCT20120718010324N71. Date of first registration 18/09/2022. Date of first sampling 20/09/2022. URL https://irct.behdasht.gov.ir/trial/65582.

## Background

Menstruation is a natural biological event that serves as an indicator of women’s health [1]. Menstrual health is “a state of complete physical, mental, and social well-being, not merely the absence of disease or infirmity, concerning the menstrual cycle” [2]. Despite menstrual disorders such as dysmenorrhea and premenstrual syndrome (PMS) significantly affecting women’s quality of life, menstrual health is often neglected in healthcare services and overlooked by public health strategies [3].

PMS and dysmenorrhea are common and recurring issues faced by women of reproductive age. PMS is a disorder characterized by a combination of physical, behavioral, and emotional symptoms that typically arise during the last week of the luteal phase, usually one week before menstruation [4]. In 1931, Robert Frank, an American physician, attributed these symptoms to ovarian dysfunction and introduced the term “premenstrual tension” at the New York Academy of Medicine. That same year, Karen Horney proposed that the syndrome might stem from sexual desires, coining the term “premenstrual syndrome [5].” Premenstrual syndrome includes a variety of symptoms, including physical signs such as bloating, breast tenderness, headaches, increased appetite, and palpitations. It also involves behavioral and psychological symptoms, including depression, irritability, fatigue, aggression, suicidal thoughts, poor concentration, mood swings, and social withdrawal [6, 7]. The causes of PMS are complex and may include hormonal, genetic, environmental, and sociocultural factors. A systematic review and meta-analysis found that the global prevalence of PMS is 48% [8]. Additionally, a systematic review conducted in Iran (2017) reported an overall prevalence rate of 70.8%. The subgroup analysis indicated a prevalence of 80.4% among high school students, 68.9% among university students, and 54.9% in the general population [9].

Primary dysmenorrhea is the recurrent lower abdominal or pelvic pain that occurs during menstruation and is not associated with any underlying pelvic abnormalities. This type of pain typically starts just before or at the onset of menstrual bleeding and can last from 8 to 72 h. It affects between 45% and 95% of women of reproductive age [10]. Although the exact cause of dysmenorrhea is not fully understood, it is thought to be associated with increased production of prostaglandins and leukotrienes [11].

Premenstrual syndrome and dysmenorrhea can negatively affect physical and social activities, interpersonal relationships, work productivity, self-esteem, and overall quality of life; it is essential to educate young women about menstrual disorders [12]. Providing support for management strategies and ensuring a multidisciplinary approach to assessment and treatment is crucial. It emphasizes the importance of identifying the causes of these disorders and pursuing effective treatment [13]. The exact causes of PMS and dysmenorrhea remain unclear. Despite exploring numerous therapeutic approaches, no definitive treatment has been identified for these conditions [14]. Various studies conducted worldwide have assessed the effectiveness of supplements and non-pharmacological methods, including exercise, dietary changes, calcium supplementation, and vitamins D, E, B6, and magnesium. However, a universally effective treatment is still absent [15, 16]. Recently, complementary medicine has gained popularity as a management option for chronic conditions like PMS and dysmenorrhea, primarily due to its cost-effectiveness, fewer side effects, and higher acceptance compared to conventional medications [17].

Curcumin is a compound known for its potent anti-inflammatory, antioxidant, and antimicrobial properties. It is derived from the rhizome of Curcuma longa, commonly known as turmeric, and has the chemical name diferuloylmethane (C21H20O6) [18]. Numerous studies have shown that curcumin provides antioxidant effects by scavenging superoxide radicals, hydrogen peroxide, and nitric oxide radicals. Its anti-inflammatory action is attributed to its ability to inhibit prostaglandins [19]. The biological activities of curcumin demonstrate its potential benefits in various areas, including anti-obesity, anti-inflammatory, anti-cancer, anti-angiogenesis, anti-diabetic, and hepatoprotective effects [20, 21].

Given the high prevalence of menstrual disorders such as PMS and dysmenorrhea among students, the limited availability of effective treatments, and the anti-inflammatory and antioxidant properties of curcumin, we decided to conduct a study to determine the effects of curcumin on PMS and dysmenorrhea symptoms among medical students in Tabriz-Iran.

## Methods

### Type of study and participants

This study was a double-blind randomized controlled trial with two parallel groups in which the researcher and the participants were unaware of group assignment. It was conducted on 62 students of Tabriz university of medical sciences.

The study’s inclusion criteria were as follows: Participants were required to have regular menstrual cycles ranging from 21 to 35 days in length. They needed to have a diagnosis of primary dysmenorrhea and premenstrual syndrome, evidenced by a Visual Analog Scale (VAS) score of ≥ 4 (indicative of moderate to severe pain) [22] and a Premenstrual Symptoms Screening Tool (PSST) score of ≥ 20 (reflecting moderate to severe symptomatology) [23]. Eligible participants were also to be within the age range of 18 to 25 years, must not exhibit any known hypersensitivity to curcumin, and should not be undergoing any pharmacological treatment for premenstrual syndrome symptoms throughout the study.

Participants were excluded if they smoked or abused alcohol, used other herbal medicines, experienced a stressful event within the past three months, suffered from any acute or chronic diseases, had a history of gynaecological disorders, underwent surgery within the last three months, were using antidepressant medications, antihistamines, barbiturates, narcotics, diazepam, amphetamines, cocaine, or taking anticoagulant drugs.

### Sample size

The sample size for this study was determined using G-Power software based on the severity of premenstrual symptoms. According to the research conducted by Khayat et al. [24], with mean values m1 = 102.06 and m2 = 71.44 (assuming a 30% reduction due to the intervention) and standard deviations SD1 = SD2 = 39.64, a significance level (two-sided α) of 0.05, and a power of 80%, the sample size was calculated to be 28 participants per group. To maintain the integrity of our study with a projected 10% dropout rate, we adjusted the sample size to 31 participants per group.

### Sampling

This study was a double-blind, randomized controlled trial designed to evaluate the effects of curcumin supplementation on the severity of PMS and dysmenorrhea among students at Tabriz University of Medical Sciences. All methods were performed in accordance with the relevant guidelines and regulations including Helsinki Declaration. After obtaining approval for the study protocol, ethical permission was confirmed by the Ethics Committee at the university. Following ethical approval and registration in the Iranian Registry of Clinical Trials (IRCT), the recruitment process commenced. The target population involved students at Tabriz University of Medical Sciences during the study period. They were screened for eligibility based on inclusion and exclusion criteria. The eligible ones were informed about the study’s objectives, procedures, and confidentiality measures. Participants received written informed consent and then completed the Premenstrual Symptoms Screening Tool (PSST) and the Visual Analog Scale (VAS). The PSST was administered during the last 5–7 days of the luteal phase (i.e., approximately one week before menstruation), consistent with the validated approach for evaluating premenstrual symptoms. Participants recorded their pain intensity using the VAS on each of the first five days of menstruation. The mean of these five scores was used to reflect overall pain severity for that cycle, in line with established recommendations for dysmenorrhea assessment [27]. No curcumin was taken in the third month to assess prolonged efficacy. Those who scored ≥ 4 on the VAS (indicating moderate to severe symptoms) [22] and ≥ 20 on the PSST (indicating moderate to severe PMS symptoms) [23] were enrolled in the study through convenience sampling.

### Randomization

Participants were randomly assigned to either the curcumin or placebo group through block randomization with block sizes of four and six, maintaining a 1:1 allocation ratio. Allocation concealment was ensured using identical, opaque, sealed containers, each sequentially numbered and containing 20 capsules for a 10-day cycle, intended for daily consumption from 7 days before to 3 days after the estimated onset of menstruation.

### Intervention

The intervention group (*n* = 31) received 500 mg curcumin capsules (Curcuma longa extract complexed with phosphatidylcholine; NOW^®^), containing a combination of curcumin and phosphatidylcholine (Phosphatidylcholine in our formulation served primarily as a bioavailability enhancer [25] and is unlikely to have independently contributed to the symptom reduction), taken once daily after a meal for 10 days during each of two menstrual cycles (7 days before to 3 days after the estimated onset of menstruation). The capsules manufactured under the supervision of a pharmacist. The control group (*n* = 31) received placebo capsules containing corn starch, identical in appearance and dosing schedule to the curcumin capsules. Curcumin or placebo capsules were administered during the first two menstrual cycles.

Participants were evaluated at four different time points: at baseline (before the intervention), 1st, 2nd, and 3rd menstrual cycles.

They received weekly reminder phone calls to ensure compliance. Each participant received a pack of ibuprofen and instructions to refrain from using other medications, along with a checklist to document any adverse effects.

### Data collection tools

The following tools were used for data collection: The sociodemographic characteristics questionnaire, the Premenstrual Symptoms Screening Tool (PSST), and the Visual Analogue Scale (VAS). Data were collected through face-to-face interviews.

### The sociodemographic characteristics questionnaire

The questionnaire collected information on age, weight, height, body mass index (BMI), educational level, and menstrual characteristics, including cycle length.

### Premenstrual symptoms screening tool (PSST)

This screening tool, developed by Steiner et al. in 2003, is designed to reflect the DSM-IV criteria for PMS and Premenstrual Dysphoric Disorder (PMDD) [26]. The PSST consists of 19 items divided into two sections. The first section includes 14 items based on psychological, physical, and behavioral symptoms. The second section consists of 5 items assessing the impact of premenstrual symptoms on daily life, such as interference with functioning in work, family, relationships, social activities, or household responsibilities. The tool uses a 4-point Likert scale (0 = not at all, 1 = mild, 2 = moderate, 3 = severe), with total scores ranging from 0 to 57. Based on the total score, symptom severity is categorized into three groups: no/mild PMS, moderate to severe PMS, and PMDD. Participants with moderate to severe PMS symptoms were involved in the study. The validity and reliability of this tool were first confirmed in Iran by Hariri et al. [23].

### Visual analogue scale (VAS)

The severity of menstrual pain was assessed using the VAS, a precise and subjective tool consisting of a 10-centimeter ruler for evaluating pain intensity. A score of 0 indicates no pain, 1–3 indicates mild pain, 4–7 indicates moderate pain, and 8–10 indicates severe pain. Participants who scored 4 or higher were included in the study. The validity and reliability of this tool have been confirmed in Iran [27].

### Side effects checklist

Participants received a checklist of potential side effects at the start of the study, which included gastrointestinal issues (nausea, bloating, diarrhea, constipation), allergic reactions (skin rashes, itching), and headaches. They were asked to report any side effects during follow-up assessments.

### Data analysis

Data were analyzed using SPSS version 26.0 with both descriptive and analytical statistics. Kolmogorov-Smirnov test was used to determine the normality of quantitative variables and all data had normal distribution. Various statistical tests assessed group homogeneity regarding sociodemographic characteristics, including the chi-square test, chi-square for trend, independent t-test, and Fisher’s exact test. An independent t-test was performed before the intervention, followed by an ANCOVA (Analysis of Covariance) after the intervention to compare the severity of dysmenorrhea and premenstrual syndrome symptoms at 1st, 2nd, and 3rd menstrual cycles. Comparisons were made across three separate cycles using ANCOVA test, controlling for pre-intervention values. A Sidak correction was applied for post-hoc pairwise comparisons to adjust for multiple testing. Figures 2 and 3 were generated to visualize the results obtained from the statistical model. All analyses were conducted based on intention-to-treat. We considered a significance level of *P* < 0.05.

## Results

**Fig. 1:**
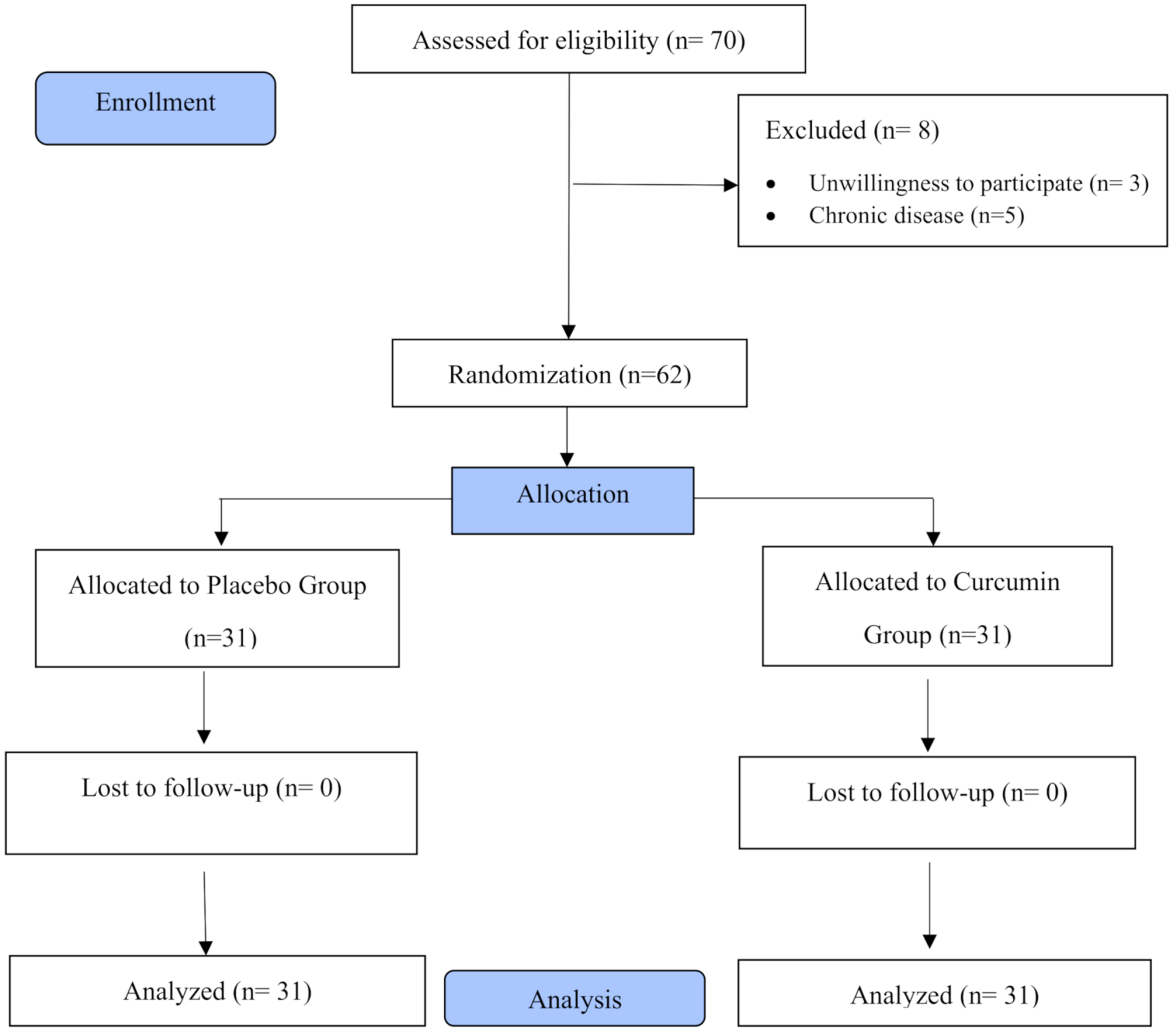
Flow chart of the study

The sampling process began in April 2023 and continued through February 2024. Out of the 70 individuals evaluated by the researcher, 62 met the required criteria for participation in the study. All 31 women assigned to each group completed the treatment period (Fig. 1).

The mean (SD) age of participants was 22.6 (1.9) in the curcumin group and 22.6 (2.1) in the placebo group. The mean (SD) menstruation age of participants was 13.0 (1.1) in the curcumin group and 13.0 (1.2) in the placebo group. The sociodemographic characteristics of the participants are outlined in Table 1; analysis indicated no significant differences between the study groups in this respect (*P* > 0.05).

**Table 1:** Socio-demographic characteristics of the participants (*n* = 62)

Characteristic	Curcumin (*n* = 31)	Placebo (*n* = 31)	*P*-value
	**Mean (SD)**	**Mean (SD)**	
**Age** (Year)	22.6 (1.9)	22.6 (2.1)	0.951^†^
**Menstruation age** (Year)	13.0 (1.1)	13.0 (1.2)	0.830^†^
**Body Mass Index (Kg/m**^**2**^)	23.2 (3.2)	22.0 (3.4)	0.192^†^
	**Number (Percent)**	**Number (Percent)**	
**Menstruation length**			0.560^†^
3–5 days	19 (61.3)	17 (54.8)	
7 days	10 (32.3)	11 (35.5)	
7 < days	2 (6.5)	3 (9.7)	
**Marital Status**			0.473^¥^
Single	25 (80.6)	28 (90.3)	
Married	6 (19.4)	3 (9.7)	
**Education**			0.863^‡^
Bachelor	22 (71.0)	22 (71.0)	
Master	5 (16.1)	4 (12.9)	
PhD	4 (12.9)	5 (16.1)	
**Father’s job**			1.000^¥^
Unemployed	0 (0.0)	1 (3.4)	
Freelance	13 (48.1)	14 (48.3)	
Employee	11 (40.7)	12 (41.4)	
Shopkeeper	1 (3.7)	0 (0.0)	
Professional	2 (7.4)	2 (6.9)	
**Mother’s job**			1.000^§^
Housewife	21 (67.7)	21 (32.3)	
Employee	10 (67.7)	10 (32.3)	
**Financial status**			0.826^‡^
Perfect	2 (6.5)	1 (3.2)	
Good	19 (61.3)	21 (67.7)	
Moderate	10 (32.3)	8 (28.5)	
Weak	0 (0.0)	1 (3.2)	
**Residency**			0.772^¥^
Private home	7 (22.6)	9 (29.0)	
Dormitory	23 (74.2)	22 (71.0)	
Relative’s home	1 (3.2)	0 (0.0)	
**Smoking**			1.000^¥^
Yes	1 (3.2)	1 (3.2)	
No	31 (96.8)	31 (96.8)	
**Alcohol**			1.000^¥^
Yes	1 (3.2)	0 (0.0)	
No	30 (96.8)	31 (100.0)	
**Allergy**			1.000^¥^
Yes	3 (9.7)	3 (9.7)	
No	28 (90.3)	28 (90.3)	
**Pain intensity during menstruation**			0.056 ^‡^
Moderate	22 (71.0)	28 (90.3)	
Severe	9 (29.0)	3 (9.7)	
**Family history of PMS**			0.642^§^
Yes	19 (51.4)	17 (45.9)	
No	18 (48.6)	20 (54.1)	
**Using Painkiller**			1.000^§^
Yes	19 (61.3)	19 (61.3)	
No	12 (38.7)	12 (38.7)	

^†^Independent t-test; ^§^Chi-square test; ^¥^ Fisher’s exact test; ^‡^ Liner by liner association

The mean (SD) of the Premenstrual Symptoms score before the intervention in the curcumin group was 52.7 (8.9), which declined to 42.6 (10.6) after the intervention; in the placebo group, it was 49.7 (9.2) before the intervention and 44.1 (8.7) after the intervention. According to the independent t-test, there was no significant difference between the study groups before the intervention (*P* = 0.204). Based on the ANCOVA test by adjusting the baseline score, the curcumin group showed a significant reduction in average score of PSST in 2nd menstrual cycle (MD: -5.2; 95% CI: -9.6 to -0.9; *P* = 0.018) compared to the placebo group (Table 2).

**Table 2:** Comparison of the mean score of premenstrual symptoms among study groups

Variable	Curcumin Mean (SD^†^) (*n* = 31)	Placebo Mean (SD^†^) (*n* = 31)	Mean Difference (95% Confidence Interval)	*P*-value
**Premenstrual Symptoms** (Score range: 0 to 57)				
Before intervention	52.7 (8.9)	49.7 (9.2)	2.9 (-1.6 to 7.5)	0.204
1st menstrual cycle	42.6 (10.6)	44.1 (8.7)	-2.9 (-7.5 to 1.6)	0.201
2nd menstrual cycle	37.7 (8.5)	42.3 (8.5)	-5.2 (-9.6 to -0.9)	0.018
3rd menstrual cycle	35.7 (9.6)	40.2 (11.0)	-5.0 (-10.3 to 0.2)	0.064

^†^ Standard Deviation

The independent t-test was used before the intervention and ANCOVA after the intervention by adjusting baseline values

The mean (SD) of the Visual Analogue Scale score before the intervention in the curcumin group was 5.4 (2.1), which declined to 4.3 (2.1) after the intervention; in the placebo group, it was 5.6 (1.9) before the intervention and 4.9 (1.7) after the intervention. According to the independent t-test, there was no significant difference between the study groups before the intervention (*P* = 0.755). Based on the ANCOVA test and by adjusting the baseline values, the curcumin group showed a significant reduction in average score of VAS in 2nd menstrual cycle (MD: -0.8; 95% CI: -1.4 to -0.1; *P* = 0.012) and 3rd menstrual cycle (MD: -0.8; 95% CI: -1.5 to -0.08; *P* = 0.029) compared to the placebo group (Table 3).

**Table 3:** Comparison of the mean score of visual analogue scale among study groups

Variable	Curcumin Mean (SD^†^) (*n* = 31)	Placebo Mean (SD^†^) (*n* = 31)	Mean Difference (95% Confidence Interval)	*P*-value
**VAS Score** (Score range: 0 to 10)				
Before intervention	5.4 (2.1)	5.6 (1.9)	-0.1 (-1.1 to 0.8)	0.755
1st menstrual cycle	4.3 (2.1)	4.9 (1.7)	-0.4 (-1.1 to 0.1)	0.159
2nd menstrual cycle	3.5 (1.9)	4.4 (1.6)	-0.8 (-1.4 to -0.1)	0.012
3rd menstrual cycle	3.4 (2.0)	4.3 (1.6)	-0.8 (-1.5 to -0.1)	0.029

^†^ Standard Deviation

The independent t-test was used before the intervention and ANCOVA after the intervention by adjusting baseline values

**Fig. 2:**
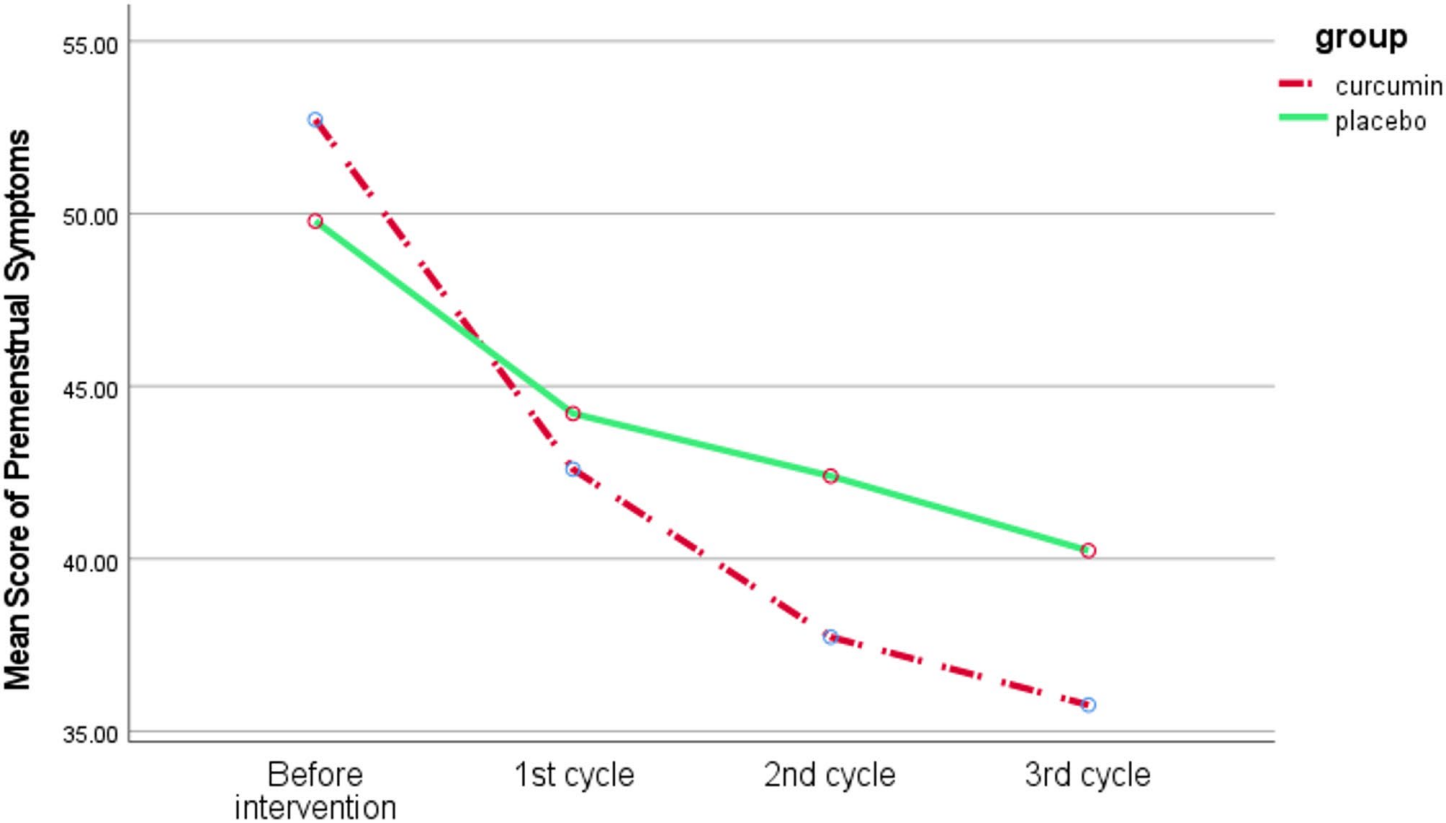
Mean score of premenstrual symptoms among study groups at baseline, 1st, 2nd, and 3rd menstrual cycles

**Fig. 3:**
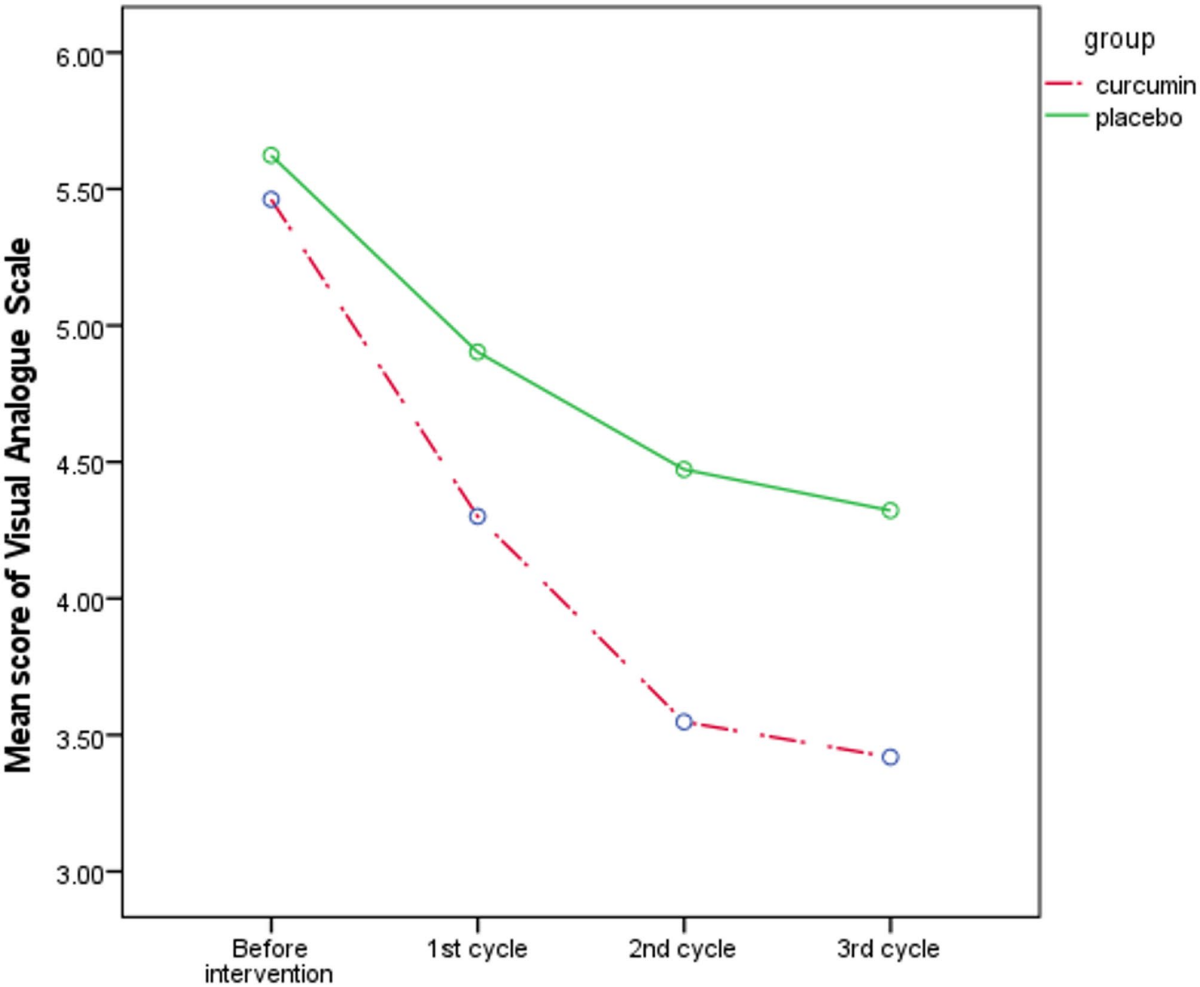
Mean score of visual analogue scale among study groups at baseline, 1st, 2nd, and 3rd menstrual cycles

The results concerning Premenstrual Symptoms and the Visual Analogue Scale are detailed following the analysis performed using ANCOVA among the study groups (Figs. 2 and 3).

None of the study groups reported adverse effects. While exact ibuprofen usage data were not recorded, participants were instructed to avoid its use unless symptoms were intolerable. There were no dropouts and all participants completed the treatment period successfully.

## Discussion

Our study demonstrated the efficacy of oral curcumin supplementation in reducing the severity of PMS and dysmenorrhea in university students in Tabriz, Iran, supporting its therapeutic potential for menstrual-related disorders.

Curcumin demonstrates its therapeutic effects primarily through its potent anti-inflammatory, antioxidative, and analgesic mechanisms. It mitigates inflammation and associated pain by inhibiting the cyclooxygenase-2 (COX-2) enzyme and decreasing prostaglandin synthesis, the critical factor in dysmenorrhea [28]. Furthermore, curcumin enhances the body’s antioxidant defense systems, effectively reducing oxidative stress, a key contributor to the pathophysiology of both PMS and dysmenorrhea [29]. While turmeric is used in local dishes, typical daily dietary intake is substantially lower than the administered dose [18], minimizing dietary interference with our study.

The findings of our study support and expand the existing evidence regarding the effectiveness of curcumin in managing premenstrual syndrome (PMS) and dysmenorrhea. The observed mean reductions in PSST and VAS scores in 2nd and 3rd menstrual cycles were statistically meaningful [22, 26]. Recent systematic reviews and randomized controlled trials (RCTs) further reinforce our results, demonstrating curcumin’s strong anti-inflammatory, analgesic, and mood-regulating properties. For example, Mawarti et al. (2024) found that curcumin reduced both the intensity and duration of dysmenorrhea while increasing vitamin D levels and decreasing serum IgE, aspartate transaminase (AST), and direct bilirubin levels in women with dysmenorrhea [30]. Similarly, Boroujeni et al. (2024) examined the mechanisms of curcumin in detail, reporting its ability to reduce oxidative stress, maintain hormonal balance, and enhance neurotransmitter levels such as serotonin and dopamine. These findings further emphasize curcumin’s potential in addressing both the physical and psychological symptoms of PMS and dysmenorrhea [28].

Recent RCTs have provided compelling evidence supporting the efficacy of curcumin. In a triple-blind trial conducted by Bahrami et al. (2021) involving 124 participants, a significant 64% reduction in dysmenorrhea pain was observed in the curcumin group, compared to a 53.3% reduction in the placebo group. These results not only emphasize the substantial impact of curcumin but also illuminate the challenges associated with distinguishing its effects from placebo responses, indicating the need for more sophisticated methodological approaches in future investigations [31]. Further corroborating these findings, a study by Talebpour et al. (2023) demonstrated that curcumin significantly reduced serum high-sensitivity C-reactive protein (hsCRP), which serves as a biomarker for systemic inflammation, thereby further validating its anti-inflammatory potential in conditions marked by elevated prostaglandin levels [32]. Karbasi et al. (2024) found that individuals with PMS and dysmenorrhea experienced increased free radical scavenging activity and antioxidant capacity after taking curcumin supplements. This evidence highlights curcumin’s potential in reducing oxidative stress-related menstrual symptoms [29].

Curcumin’s antidepressant effects, mediated by the regulation of serotonin and dopamine, provide a therapeutic avenue for alleviating psychological distress associated with PMS [28]. Comparisons with pharmaceutical treatments indicate that curcumin may be an effective and safer alternative [33]. Khayat et al. (2015) found that curcumin treatment significantly reduced the severity of PMS, which they attributed to its serotonergic and anti-inflammatory properties. The study observed notable improvements in mood and physical symptoms, further validating our findings [24]. Also, Putri et al. (2023) demonstrated that curcumin’s COX inhibition is comparable to that of non-steroidal anti-inflammatory drugs (NSAIDs) commonly prescribed for dysmenorrhea. It suggests curcumin could be a promising option for those seeking natural remedies with fewer side effects [34]. Curcumin may offer comparable benefits to NSAIDs such as ibuprofen in managing dysmenorrhea while avoiding gastrointestinal side effects [35]. Furthermore, its mood-stabilizing effects may provide broader PMS symptom relief compared to pharmacologic agents targeting only pain [36].

While previous clinical studies have examined the effects of curcumin on premenstrual syndrome and dysmenorrhea, our study offers several novel contributions. Unlike earlier trials [24, 31], which primarily assessed short-term effects and used standard curcumin formulations, we evaluated the impact of a phosphatidylcholine-complexed curcumin supplement, which is known to enhance bioavailability. Furthermore, our trial extended over three menstrual cycles, allowing us to explore both immediate and residual effects following cessation of the supplement. This prolonged follow-up period has been largely overlooked in previous randomized controlled trials. Additionally, our study population consisted exclusively of Iranian university students, a specific demographic with a high prevalence of menstrual disorders, providing valuable culturally contextualized data. Importantly, although mechanistic hypotheses—such as anti-inflammatory and serotonergic effects—have been proposed in the literature [37], our findings add empirical human-level data showing significant premenstrual symptom reduction, supporting the continued clinical investigation of curcumin in reproductive health.

### Strengths and limitations

This study adheres to all principles of clinical trials, including random allocation and allocation concealment. Using standardized instruments, we ensured accurate and consistent evaluation of outcomes, which enhanced the reproducibility of our findings. Furthermore, the intervention extended over multiple menstrual cycles, allowing us to observe sustained effects over time rather than just short-term changes. Our research focused on a young population, providing valuable insights into the effects of curcumin on premenstrual syndrome (PMS) and dysmenorrhea in a demographic that is often affected by these conditions. Although the study showed promising results, it had some limitations. Variations in diet, stress levels, and physical activity among participants could impact the severity of symptoms, indicating a need for more stringent controls in future research. Additionally, by concentrating on a young and healthy group, it remains uncertain whether these findings are applicable to women with coexisting conditions or those in different age groups. Although we did not record the exact number of ibuprofen tablets taken, we recognize that future studies could benefit from more detailed analgesic usage logs to better quantify adjunct medication effects.

## Conclusion

This study highlights the increasing evidence that curcumin is an effective natural treatment for PMS and dysmenorrhea. Further research into dose optimization, combination therapies, and long-term effects will strengthen its position in clinical practice.

## Data Availability

The data used to support the findings of this study can be obtained from the corresponding author upon request.

## Abbreviations

ANCOVA: Analysis of Covariance
hsCRP: Serum high-sensitivity C-reactive protein
MD: Mean difference
NSAIDs: Non-steroidal anti-inflammatory drugs
PMS: Premenstrual syndrome
PSST: Premenstrual Symptoms Screening Tool
RCT: Randomized controlled trial
VAS: Visual Analogue Scale
